# Prognostic awareness and prognostic information preferences among advanced cancer patients in Kenya

**DOI:** 10.4102/phcfm.v16i1.4288

**Published:** 2024-04-11

**Authors:** Hussein Elias, Semra Ozdemir, Joann Bairavi, Emmah Achieng, Eric A. Finkelstein

**Affiliations:** 1 Department of Family Medicine, Moi University School of Medicine, Eldoret, Kenya; 2 Academic Model Providing Access to Healthcare (AMPATH), Eldoret, Kenya; 3 Lien Centre for Palliative Care, Duke-NUS Medical School, Singapore, Republic of Singapore; 4 Department of Population Health Sciences, Duke Clinical Research Institute, Duke University, Durham, United States of America; 5 Moi Teaching and Referral Hospital, Eldoret, Kenya; 6 Duke Global Health Institute, Duke University, Durham, United States of America

**Keywords:** prognosis, prognostic awareness, prognostic information preferences, advanced cancer, Kenya

## Abstract

**Background:**

Cancer is the third leading cause of death in Kenya. Yet, little is known about prognostic awareness and preferences for prognostic information.

**Aim:**

To assess the prevalence of prognostic awareness and preference for prognostic information among advanced cancer patients in Kenya.

**Setting:**

Outpatient medical oncology and palliative care clinics and inpatient medical and surgical wards of Moi Teaching and Referral Hospital (MTRH) in Eldoret, Kenya.

**Methods:**

The authors surveyed 207 adults with advanced solid cancers. The survey comprised validated measures developed for a multi-site study of end-of-life care in advanced cancer patients. Outcome variables included prognostic awareness and preference for prognostic information.

**Results:**

More than one-third of participants (36%) were unaware of their prognosis and most (67%) preferred not to receive prognostic information. Increased age (OR = 1.04, 95% CI: 1.02, 1.07) and education level (OR: 1.18, CI: 1.08, 1.30) were associated with a higher likelihood of preference to receive prognostic information, while increased symptom burden (OR= 0.94, CI: 0.90, 0.99) and higher perceived household income levels (lower-middle vs low: OR= 0.19; CI: 0.09, 0.44; and upper middle- or high vs low: OR= 0.22, CI: 0.09, 0.56) were associated with lower odds of preferring prognostic information.

**Conclusion:**

Results reveal low levels of prognostic awareness and little interest in receiving prognostic information among advanced cancer patients in Kenya.

**Contribution:**

Given the important role of prognostic awareness in providing patient-centred care, efforts to educate patients in Kenya on the value of this information should be a priority, especially among younger patients.

## Introduction

Cancer is the third leading cause of death by non-communicable disease in Kenya and incidence rates are increasing, up 37 000 from 2012 to 47 887 new cases in 2018.^1,2,3^ Despite rising incidence, no information exists regarding prognostic awareness or preferences regarding prognostic information among patients with advanced cancer in Kenya. Based on evidence from other African countries, many advanced cancer patients in Kenya are likely to be unaware of their prognosis or not inclined to receive prognostic information.^4,5^

Prognostic awareness can be defined as awareness of disease incurability and shortened life expectancy^6^ is associated with more frequent end-of-life discussions,^7^ more patient-centric care,^8,9^ earlier palliative support, fewer unwanted resuscitations^10^ and increased shared decision-making.^11^ Despite these benefits, prognostic awareness remains low among patients with advanced cancer globally.^5,12^ Patient preference for receiving prognostic information is thought to have increased in recent years as informed decision-making has become more common in medical settings.^13^ Nevertheless, studies show mixed results with some suggesting patients prefer not to receive prognostic information.^14,15^

We therefore aimed to examine prognostic awareness and preference for prognostic information among patients with advanced cancer treated at a single cancer centre in Kenya. We also assessed the relationship between prognostic awareness and preference for prognostic information and patient factors. Based on prior literature, we hypothesised that greater prognostic awareness and preference for receiving prognostic information would be associated with younger age,^13,16^ higher education,^14,15^ higher income^16,17^ and higher symptom burden.^18,19^

## Research methods and design

### Study design

Data for this study were collected as part of the Asian and African Patient Perspectives Regarding Oncology Awareness, Care, and Health (APPROACH) study, a multi-country cross-sectional study of end-of-life care among advanced cancer patients.

### Setting

Data for the Kenya site were collected at Moi Teaching and Referral Hospital (MTRH), Eldoret, a level 6 hospital offering specialised oncological and palliative services with a catchment area of 24 million residents in Western Kenya, Eastern Uganda and South Sudan.

### Study population and sampling strategy

**FIGURE 1:**
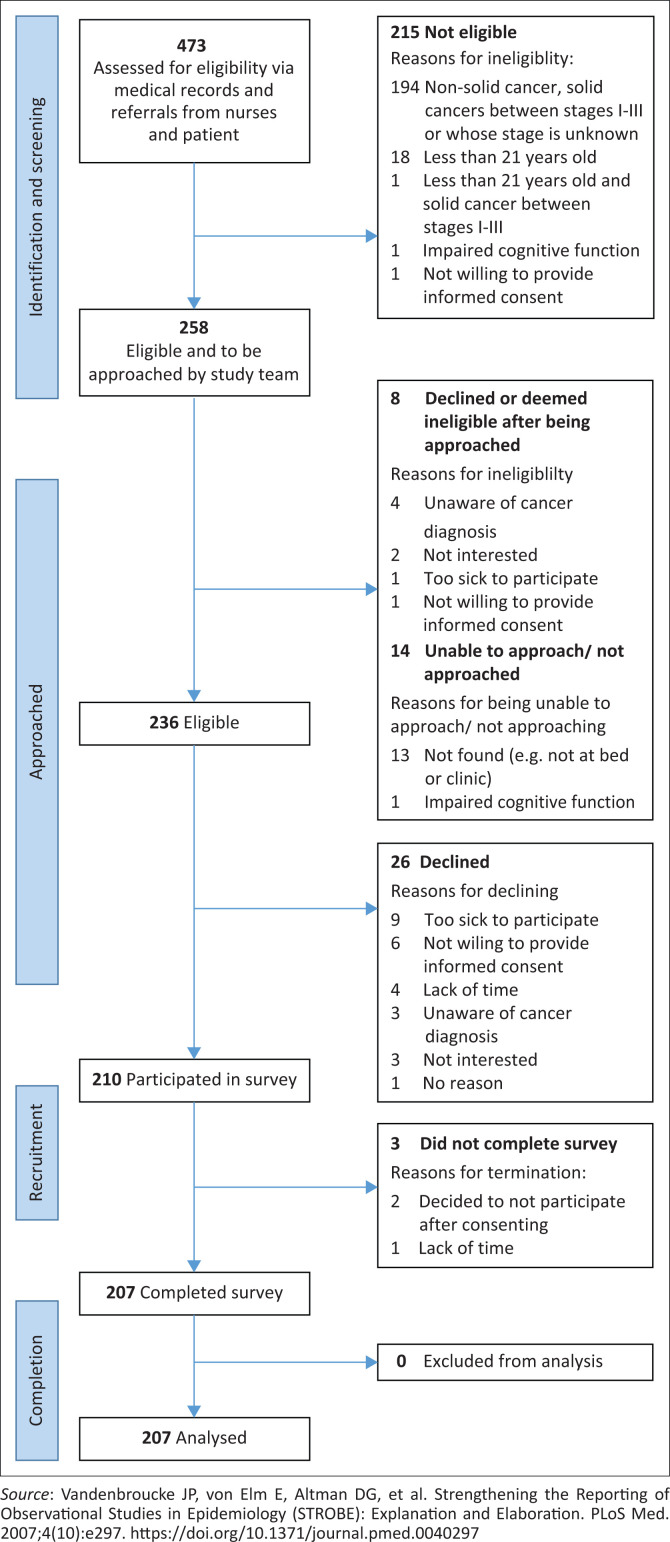
STROBE participant flow diagram.

Eligible participants were: (1) ≥ 21 years old, (2) diagnosed with Stage IV solid cancer and (3) able to understand English and (4) seeking treatment at MTRH during the study period. Consistent with the multisite APPROACH protocol, we aimed to recruit a sample of 200 participants.^20,21^ To obtain this sample size, 473 patients were assessed for eligibility via daily screening of medical records of outpatients seen at the medical oncology and palliative care departments and inpatient medical and surgical wards and referrals from nurses and patient. Of the pool of patients, 266 were deemed ineligible based on medical record review or after being approached for the study or declined to participate. The remaining 207 patients were recruited for face-to-face interviews conducted at MTRH between October 2021 and February 2022. The STROBE participant flow diagram can be found in Figure 1^22^. The Strengthening the Reporting of Observational Studies in Epidemiology (STROBE) checklist can be found in Online Appendix 1.

### Measures

The survey questionnaire comprised validated scales and items developed by APPROACH study investigators in consultation with oncologists and research faculty at participating centres.^23^

#### Prognostic awareness

To assess prognostic awareness, participants were asked: ‘Do you know the current stage (i.e. severity) of your cancer?’. As all respondents were advanced cancer patients, ‘Early stage (Stage I, II, or III)’ and ‘don’t know’ responses were categorised as ‘unaware’ while ‘Advanced cancer (Stage IV)’ responses were categorised as ‘aware’.

#### Patient preference for prognostic information

Patients were asked, ‘Would you like to know how long you are likely to live under various treatment options?’ A dichotomous variable for patient preference was created with a value of 1 for patients indicating, ‘yes’ in ‘general or specific terms’ and 0 for patients indicating ‘no’ or ‘not sure’.

#### Participants’ characteristics

Patients’ age was extracted from patient medical records; all other variables were based on self-report. Participants reported years of education, perceived household economic status (0: low, 1: lower-middle, 2: upper middle or high) and how long they had known about their cancer diagnosis (0: <1 year, 1: 1 to 3 years, 2: > 3 years). Symptom burden was assessed using questions adapted from the Functional Assessment of Chronic Illness Therapy – Palliative Care instrument (FACIT-Pal) (Version 4).^24^ Examples of symptoms assessed included pain, shortness of breath and unintentional weight loss. The symptoms were scored on a 5-point Likert scale ranging from ‘0: not at all’ to ‘4: very much’. Scores were then summed (total score range: 0 to 40) with higher scores indicating greater symptom burden.

### Data analysis

We first summarise participants’ characteristics with mean and standard deviations (s.d.) for continuous variables and frequencies and percentages for categorical variables. We then fit two binary logistic regression models to assess the association between participant characteristics and: (1) prognostic awareness and (2) preference for prognostic information. The dependent variables were prognostic awareness (0 = unaware, 1 = aware) and preference for prognostic information (0 = no or not sure; 1 = yes). Independent variables for both models were age, years of education, socioeconomic status and symptom burden.

We also conducted a post hoc estimation of the variance inflation factor (VIF) to assess multicollinearity between the independent variables.^25^ All analyses were conducted using Stata version 15.1.

### Ethical considerations

Ethics approval was obtained from the National University Singapore-Institutional Review Board (NUS-IRB LB-15-319) and the Moi Institutional Research and Ethics Committee (IREC/2021/27). Trained interviewers obtained written informed consent from all participants prior to the survey. Only the trained interviewers and study team at MTRH had access to participants’ confidential information. Participants were assigned a unique identification number and only de-identified data were collected and analysed.

## Results

### Participants’ characteristics

Table 1 presents sample characteristics. Respondents ranged in age from 21 to 85 with a mean age of 55 (SD = 15.4) and an average of 8.5 (SD = 4.6) years of education. Slightly more respondents were female (57%) than male and most were married (76%). Perceived household economic status varied among participants with 37% indicating low, 38% indicating lower-middle and a quarter indicating upper-middle or high-income. Most participants (53%) had known of their cancer diagnosis for 1 to 3 years. Participants reported a mean symptom burden score of 13.9 (SD = 7.8; range: 0 to 37).

**TABLE 1:** Participant Characteristics *N* = 207.

Participant characteristics	Mean	s.d.	Range	*n*	%
**Age**	55.3	15.4	21-85	-	-
**Gender**					
Male	-		-	89	43
Female	-	-	-	118	57
**Marital status**					
Married	-	-	-	158	76
Not married	-	-	-	49	24
**Years of education** ‡	8.5	4.6	0–19	-	-
**Socioeconomic Status** §					
Low income	-	-	-	76	37
Lower middle income	-	-	-	79	38
Upper middle/high income	-	-	-	51	25
**Years since diagnosis**					
< 1	-	-	-	42	20
1 to 3	-	-	-	110	53
> 3	-	-	-	55	27
**Symptom burden**	13.9	7.8	0-37		
**Dependent variables**					
Prognostic awareness					
Aware	-	-	-	133	64
Unaware	-	-	-	74	36
Preference for Prognostic Information†					
Yes	-	-	-	64	33
No/unsure	-	-	-	132	67

Note: Due to rounding, percentages may not add up to 100%.

† , *n* = 196; 11 participants did not respond to the question on preferences for prognostic information;

‡ , *n* = 206;

§ , *n* n = 206.

### Prognostic awareness

Nearly two-thirds of participants were aware of their prognosis (64%) (Table 1). When we examined participants’ characteristics associated with prognostic awareness in the logistic regression model, we found no significant associations (Table 2).

**TABLE 2:** Associations between prognostic awareness and participant characteristics, *N* = 205.

Participant characteristics	Odds ratio	95%, CI
Age	0.99	0.97, 1.01
Years of education	0.99	0.93, 1.07
**Socio-economic status (ref: low income)**		
Lower-middle income	0.82	0.42, 1.63
Upper-middle or high income	0.73	0.34, 1.57
Symptom burden	1.01	0.97, 1.05

CI, confidence interval.

### Preference for prognostic information

One-third (33%) of participants indicated a preference to receive prognostic information (Table 1). When we examined participants’ characteristics associated with preference for prognostic information in the logistic regression model, we found significant associations with all participant characteristics (Table 3). Contrary to our hypotheses, increased age was associated with a higher likelihood (odds ratio [OR] = 1.04, 95% confidence interval [CI]: 1.02, 1.07) and increased symptom burden was associated with a lower likelihood (OR = 0.94, CI: 0.90, 0.99) of preference to receive prognostic information. Also contrary to our hypotheses, participants reporting higher perceived household income levels (lower-middle vs. low: OR = 0.19; CI: 0.09, 0.44 and upper middle or high vs. low: OR = 0.22, CI: 0.09, 0.56) were less likely to prefer receiving prognostic information. Supporting our hypothesis, participants reporting higher education levels were more likely (OR: 1.18, CI: 1.08, 1.30) to prefer prognostic information (Table 3). We did not observe multicollinearity in either of the multivariable models (VIF < 2).

**TABLE 3:** Associations between patient preferences for prognostic information and participant characteristics (*N* = 195).

Participant Characteristics	Odds Ratio	95%, CI
Age	1.04**	1.02, 1.07
Years of education	1.18**	1.08, 1.30
**Socio-economic status (ref: low income)**		
Lower-middle income	0.19**	0.09, 0.44
Upper-middle or high income	0.22**	0.09, 0.56
Symptom burden	0.94*	0.90, 0.99

CI, confidence interval.

* , *p* < 0.05;

** , *p* < 0.01.

## Discussion

The primary aim of this study was to examine the prevalence of prognostic awareness and prognostic information preference among advanced cancer patients in Kenya. We also assessed associations between these outcomes and various participants’ characteristics. More than one-third of participants (36%) were unaware of their prognosis (defined as current stage [i.e. severity] of cancer), and the majority of participants (67%) preferred not to receive prognostic information. Our findings related to prevalence of prognostic awareness are consistent with other studies of patients in Africa.^5^ Although our study did not investigate causal factors behind the low levels of prognostic awareness, prior research has suggested the paternalistic nature of the medical system in which patients are often not included as decision-makers, which may have played a role in low prognostic awareness.^26,27^ However, our results suggest most patients also prefer not to receive prognostic information.

To understand factors influencing prognostic awareness, we examined the relationships between awareness and preference for prognostic information and observable participants’ characteristics. Prognostic awareness was not significantly associated with any of the patient factors examined; however, preference for prognostic understanding was strongly associated with patient factors. In line with prior literature, participants in our study reporting higher education levels were more likely to prefer to receive prognostic information.^15^ These findings are consistent with the notion that individuals with higher education levels tend to have better health literacy.

Contrary to prior studies, we found two generally disenfranchised groups, older adults and those reporting lower household income levels were more interested in receiving prognostic information than their counterparts. Older adults tend to have more emotional stability and to be more accepting of their situation,^28^ which may explain why they feel more comfortable requesting and receiving prognostic information.^29^ Although potential reasons for differences by perceived household income level are less obvious, it may be that individuals with lower income levels are more motivated to understand their illness trajectory so they have a better sense of related financial consequences, which may disproportionately impact their households.^30^ Lastly, we found higher symptom burden was associated with a lower likelihood of preference for prognostic information, perhaps suggesting patients with increased symptom burden are weary of receiving bad news.

### Strengths and limitations

The strength of this study lies in its examination of patient prognostic awareness and preference for receiving prognostic information in a country in Africa (Kenya) where little information currently exists. This study has several limitations. One limitation was that the study was conducted in a single site in Kenya, and thus results may not be generalisable to other sites in Kenya or Africa. Likewise, the focus on a single condition (i.e. advanced cancer) and missing data (~5%) are additional limitations. Most importantly, this study evaluates correlations and thus cannot identify causal reasons behind the low levels of prognostic awareness and preference for receiving prognostic information. Future studies should explore causal factors related to low prognostic awareness and information preference among advanced cancer patients in Africa.

## Conclusion

Our results reveal low levels of prognostic awareness and little interest in receiving prognostic information among advanced cancer patients in Kenya. Given the important role of prognostic awareness in providing patient-centred care, efforts to educate patients in Kenya on the value of this information should be a priority, especially among younger patients who were less likely to prefer prognostic information in our study. Interventions to address these concerns might include public health campaigns on the value of informed decision-making, provider training in health communication^31^ and protocols requiring informed consent^32^ and patient and provider education on advance care planning. Future research should test the effectiveness of these interventions in improving prognostic awareness.
